# Analysis of available animal testing data to propose peer-derived quantitative thresholds for determining adequate surveillance capacity for rabies

**DOI:** 10.1038/s41598-023-30984-3

**Published:** 2023-03-09

**Authors:** Faisal S. Minhaj, Sarah C. Bonaparte, Cassandra Boutelle, Ryan M. Wallace

**Affiliations:** Poxvirus and Rabies Branch, Division of High Consequence Pathogens and Pathology, Centers for Disease Control and Prevention, Atlanta, USA

**Keywords:** Viral infection, Ecological epidemiology

## Abstract

Historical targets for country-level animal rabies testing volumes were abandoned due to ethical and welfare concerns, and interpretation challenges of testing healthy animals. To-date, no quantitative threshold has been established for evaluating adequate surveillance capacity specific to suspected rabid animals. The purpose here is to establish quantitative testing thresholds for rabies suspected animals to assess a country’s rabies surveillance capacity. Animal rabies testing data was obtained from official and unofficial rabies surveillance platforms from 2010 to 2019 and supplemented with official country reports and published literature. Testing rates were determined for all-animal and domestic animals, and standardized per 100,000 estimated human population; the domestic animal rate was also standardized per 100,000 estimated dog population. There were 113 countries that reported surveillance data eligible for analysis. Countries reporting the most data were under WHO categories as having endemic human rabies or no dog rabies. The annual median all-animal testing rate for all countries was 1.53 animals/100,000 human population (IQR 0.27–8.78). Three proposed testing rate thresholds are an all-animal rate of 1.9 animals/100,000 humans, a domestic animal per human rate of 0.8 animals/100,000 humans, and a domestic animal per dog rate of 6.6 animals/100,000 dogs. These three peer-derived rabies testing thresholds for passive surveillance can be used to facilitate assessment of a country’s rabies surveillance capacity.

## Introduction

Rabies is an under-diagnosed, under-reported disease and accurate data on global human deaths, access to vaccines, and animal disease incidence is limited^1,2^. Rabies virus is classified under the lyssavirus genus and endemic to every continent except Antarctica and Australia. The disease is responsible for approximately 59,000 deaths globally every year and is nearly 100% fatal in those who do not receive post-exposure prophylaxis (PEP)^1^. Among dog mediated rabies virus variant (DMRVV) endemic countries, rabies is often listed as a priority zoonotic disease for implementing control measures^3,4^. Despite evidence of country interest in improving rabies control measures, functional rabies systems for case detection remain elusive; fewer than 10% of estimated rabies deaths are detected and fewer than one-third of World Health organization (WHO)-member-countries report these to the WHO’s Global Health Observatory (GHO). While controlling rabies remains a priority for many dog rabies endemic countries’ governments, the lack of visibility to the true burden of disease portends meaningful domestic and international investment and support^2,5^.

The WHO has committed to ending dog-mediated human rabies deaths as a public health problem. In 2021, the WHO released the updated Neglected Tropical Disease (NTD) Roadmap, which sets global targets to combat NTDs and support the United Nations (UN) Sustainable Development Goals. Rabies is the only zoonotic disease currently designated with a formal control goal, with the key indicator of success being the number of countries that are recognized as “free from dog-mediated human rabies deaths”. The World Organization for Animal Health (WOAH), the WHO, and the Food and Agricultural Organization of the United Nations (FAO) have a similar goal, with the ambitious target of the year 2030 (ZB30)^6^. Two programs have been developed to establish criteria to recognize this accomplishment. WHO has established the validation program^7,8^ which establishes a formal recognition program for which countries can apply. WOAH also has an established program of self-declaration of the elimination of DMRVV. Both programs require that a country documents that they have established the “presence of high-quality surveillance,” which is defined according to the WOAH terrestrial animal code as “the systematic ongoing collection, collation, and analysis of information related to animal health and the timely dissemination of information so that action can be taken”^9^. However, this definition does not contain objective, quantitative parameters upon which to gauge adequate surveillance.

National rabies control efforts vary by country and resource availability. Passive public health rabies surveillance defined and recommended by the WHO and WOAH for the establishment of an effective rabies surveillance program consists of evaluating and testing suspected rabid animals that are involved in human or domestic animal exposures^10–12^. This type of surveillance remains the primary element of any rabies control program and is crucial to achieving the tripartite goal of ZB30. Active surveillance is based on targeted sampling of animals that act abnormally, found dead, are gathered as roadkill, or otherwise have a pre-determined rationale for rabies testing; this can be considered in instances where passive surveillance does not provide adequate coverage due to limitations of geography or human habitation^13^. Historical recommendations have provided quantitative targets for animal rabies testing volumes to achieve such surveillance standards ranging from 0.01 to 0.02% of the total dog population, which was thought to enable detection of 5–10% of rabies cases in an enzootic setting. This approach was abandoned in 2013 due to many countries testing healthy, asymptomatic dogs to reach target testing levels. Testing animals which have no suspicion for rabies virus infection led to concerns about the ethics, welfare, and data interpretation of these surveillance programs^14,15^. Current WOAH surveillance standards imply that passive surveillance efforts should only be conducted on animals that have a clinical suspicion for rabies. The low overall prevalence of rabies and limited disease detection window make testing of healthy animals an ill-advised approach to surveillance.

Appropriate testing of sick animals improves the probability of disease detection and is cost-effective and lifesaving when used concurrently with public health programs under an integrated bite case management approach^16,17^. While historical establishment of quantitative testing thresholds was misused and subsequently abandoned, the lack of such thresholds has now led to inconsistent interpretation of the adequacy of rabies surveillance programs and lack of clarity for surveillance system operational and capacity goals. Development of empirical surveillance thresholds strictly applied to the testing of clinically suspicious animals would provide national rabies programs with the ability to better plan surveillance approaches, scale up surveillance capacities, and improve consistency in the way countries are evaluated for official rabies control status designations.

Overall, there are multiple factors that need to be considered when examining country capacity for operating an adequate rabies surveillance system that meets the WOAH and WHO expectations. Through a global landscape review of publicly available rabies testing data, this study proposes a quantitative rabies testing threshold for clinically suspicious animals that can be used to assess a country’s rabies surveillance capacity.

## Methods

To supplement the limited publicly available information on rabies risk, the US Centers for Disease Control and Prevention (CDC) performs an annual country-by-country qualitative assessment of rabies risks and protective factors. The results of this assessment are released annually in an open-access database of core metrics consisting of the presence of lyssaviruses (specifically canine or wildlife rabies virus variants, or other bat lyssaviruses), access to rabies immunoglobulins and vaccines, rabies surveillance capacity and canine rabies control capacity^18^. The analysis presented here builds upon the current CDC evaluation and specifically examines publicly available data to better inform the parameter of rabies surveillance capacity. This study found publicly available data regarding rabies animal testing by species, described testing practices in relation to the country’s human and dog populations, as well as by their stage of DMRVV control (defined by WHO), and used this data to calculate a surveillance testing threshold for DMRVV endemic countries.

Data sources were categorized into four tiers, with the order reflecting the preference for selecting the most appropriate data for the purposes of this analysis. Tier 1 data sources were considered to be the preferential data source and included any official government data submitted to a Regional or International data repository. Official data repositories included the WHO GHO, Pan-American Health Organization Regional Information System for Epidemiologic Surveillance of Rabies (PAHO SIRVERA), and the European Rabies Bulletin. Tier 1 data sources also included official country reports found through literature search, so long as they were publicly available. Tier 2 data sources consisted of published reports in peer-reviewed literature or on a ministry of health or agriculture site that includes data from the entire country, as well as unofficial data repositories (e.g., Global Alliance on Rabies Control (GARC) Rabies Epidemiologic Bulletin). Tier 3 data consisted of one-time cross-sectional studies or studies describing sub-national testing activities and which could not be reliably extrapolated to an entire country. Tier 4 data sources include any resource not captured in the previous criteria that were obtained during literature searches. The primary data search was conducted in September 2021, with an update in September 2022. Only Tier 1 and Tier 2 data sources were included in the evaluation of animal testing rates. If multiple data sources contained conflicting testing rates, we prioritized data from surveillance repositories, then reports from ministries of health or agriculture, and, finally, peer-reviewed publications.

For Tier 1 data (i.e., surveillance repository), data was included in this study if it described rabies testing conducted between the years 2010 and 2019. As political, economic, and epidemiologic factors directly influence the reliability and transparency of surveillance system data, we decided that a ten-year limit would capture any year-to-year variation in data and better characterize current passive surveillance practices. Additionally, the cutoff of 2019 was chosen so that the effects of the COVID-19 pandemic on rabies surveillance capacity would not affect this comprehensive evaluation and would account for lag time in reporting to Tier 1 data sources^19,20^. This study assumed data from these surveillance repositories is entered secondary to passive surveillance systems. If data was known to be from active surveillance activities, it was removed from analyses.

For Tier 2 data (i.e., peer-reviewed publications), certain publications presented aggregated testing data that included years prior to the Tier 1 cutoff (i.e., 2010). To increase inclusivity of eligible data and keep the findings from this evaluation representative of current practices, eligible data must have had an end year ≥ 2012, regardless of the starting year of data (Table S1). The literature search was conducted on PubMed, Scopus, and Google for “rabies” AND “[country name]” from 2010 to December 2021. “Publicly available” was defined as any result appearing in PubMed or Scopus, or within the first three pages of a Google search. Exceptions to the first three pages were made for similar country names (e.g., Guinea, Congo). The first 10% of Spanish- and French-speaking countries were also searched for “rabia” and “raj,” respectively, to potentially capture any other sources of surveillance data. However, after no additional data was found, this was discontinued. If an article or resource quantifying animal testing capacity within these criteria was not found, the country was deemed to not have readily available data for analysis.

For any countries that were part of the surveillance threshold calculation for DMRVV endemic countries, the preferred tiered data was compared to all other data sources. For one country (i.e., Brazil), there was a notable lack of dog testing data and known discrepancies in data reporting between their two reporting systems (i.e., SINAN, SIRVERA)^21^. In this situation, a median rate was calculated between a Tier 1 and Tier 3 data source. No other such discrepancies were noted. The type of surveillance (active or passive) was noted for each data source; we assumed passive surveillance with Tier 1 data unless compelling evidence existed to display that this was not the case. A strictly active surveillance program was excluded from all analyses. A summary of overall testing practices was performed and standardized according to the number of years each data source contained.

As evaluations of rabies testing rates spanned over multiple years, population estimates were obtained to reflect the most recent year in the available data. Three separate testing rates were calculated and standardized based on the human population within the country: [1] All animal, [2] Domestic animal, and [3] Wildlife. There are different social and cultural behaviors that affect the human to dog ratio and interactions between people and animals. These differences can impact the susceptibility of dogs to rabies virus infection and the likelihood of human interactions with rabid animals. Therefore, we additionally calculated country testing rates standardized by the estimated dog population, to provide an additional indicator value of adequate surveillance capacity. Estimated dog populations were obtained from a previous study^22^. This resulted in up to four calculated rabies testing rates per country, depending upon available data.

Equation 1: All-animal per human testing rate (AAHR)

1 $$\frac{Average\,number\,of\,all\,animals\,tested/year}{{Estimated\,human\,population}} \times 100,000$$

Equation 2: Domestic animal per human testing rate (DAHR)

2 $$\frac{Average\, number\, of\, domestic\, animals\, tested/year}{{Estimated\, human\, population}} \times 100,000$$

Equation 3: Domestic animal per dog testing rate (DADR)

3 $$\frac{Average\, number\, of\, domestic \,animals\, tested/year}{{Estimated \,dog\, population}} \times 100,000$$

Equation 4: Wildlife per human testing rate (WHR)

4 $$\frac{Average\, number \,of\, wildlife\, animals\, tested/year}{{Estimated \,human\, population}} \times 100,000$$

The WHO rabies epidemiologic Status is divided into five categories in escalating levels of dog rabies control: [1] Endemic dog-transmitted human rabies, [2] Endemic dog rabies, [3] Sporadic dog-transmitted rabies, [4] Controlled dog rabies, and [5] No dog rabies. The WHO Status was established based on existing data and expert knowledge to help better define the level of rabies control for each country^23^. In addition to these five WHO Statuses, countries in Status [5] were further sub-categorized into [5a] (rabies virus free), and [5b] (wildlife rabies enzootic) based on CDC’s wildlife rabies status; the CDC rabies status was also used for any country without a WHO Status (n = 11)^24^. Average testing rates for the aforementioned equations were calculated for each WHO Rabies Status category, treating each country as an equally weighted value in the rate calculation. Only descriptive analyses were conducted to describe surveillance and testing data, as data quality was not deemed acceptable for multi-variable statistical analysis and testing rates were heavily left-skewed. Data is presented as median and IQR as the data was noted to not reflect a parametric distribution.

### Ethics approval

This activity was reviewed by CDC and was conducted consistent with applicable federal law and CDC policy. (See e.g., 45 C.F.R. part 46, 21 C.F.R. part 56; 42 U.S.C. §241(d); 5 U.S.C. §552a; 44 U.S.C. §3501 et seq.) The views and opinions of the manuscript are of the authors alone and do not represent those of CDC or any other federal agency.

## Results

**Figure 1:**
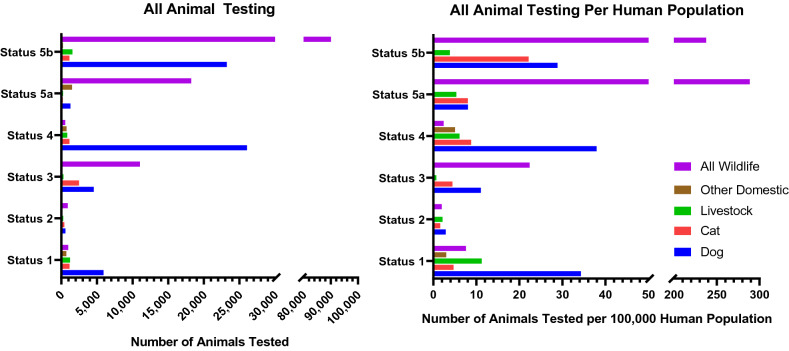
Animal testing rates stratified by WHO Rabies Status. An increase in Rabies Status from 1 to 5 is representative of a higher degree of dog rabies control. The x-axis is segmented so that lower testing numbers of animals can be visualized.

A total of 240 searches (one per country or territory) were conducted on each database to evaluate rabies surveillance. Data was found for a total of 127 (52.9%) countries and territories (Table S1). For these 127 countries, the median number of animals tested annually was 125.5 with a significant left skew. There were 98 countries which differentiated the type of animals that were tested (Fig. 1). Overall, at least 53 different species were reported, with a median of 7.5 (IQR 3–13) species reported per country tested over the study period. The most common species tested were dogs at 63,456 average tests per year (32.1% of total testing data), with 88.8% of countries reporting at least one test in a dog over the study period. Most dogs were tested by three countries (Mexico, United States, Brazil) totaling 49,334 (77.7%) of the dogs tested per year. Among the other domestic animals tested, cats were the most common at 29,700 average tests per year (71.3% from the United States), with the number of countries reporting testing in cats at 77.6% over the study period. Among the 19 countries where bats are known to carry rabies virus, 14 (73.7%) reported testing bats annually. However, 50 total countries (51.0%) reported testing bats, likely for other non-rabies lyssaviruses, with 29,492 average tests per year during the study period (90.5% from the US and Brazil). Other common wildlife species tested were foxes (42,010/year), raccoons (14,483/year), and raccoon dogs (1,926/year).

### Eligible studies

**Figure 2:**
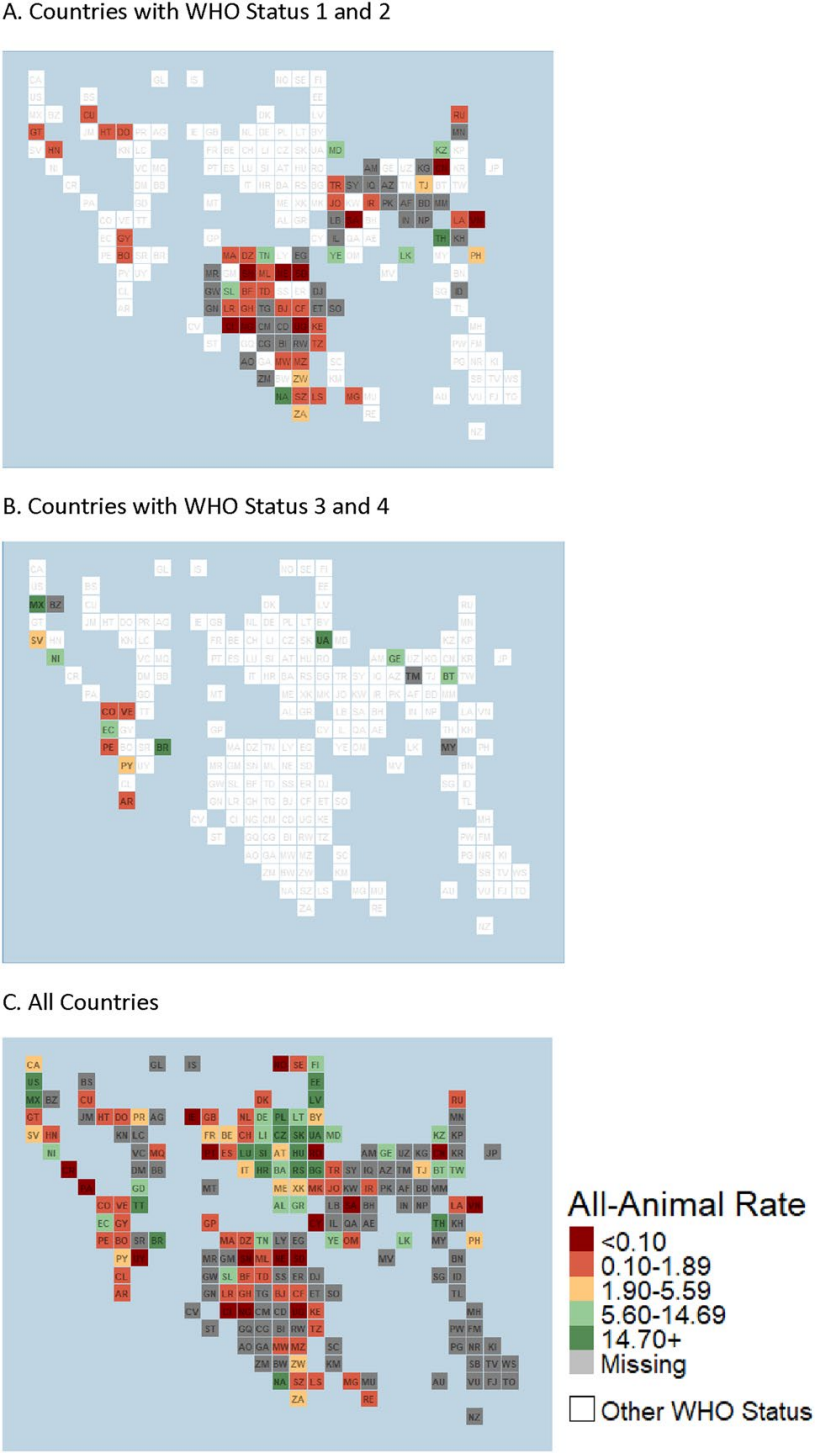
All-animal Testing Rate/100,000 Human Population. Countries are represented by their official two letter ISO 3166 alpha-2 (https://www.iban.com/country-codes), in equal tiles such that countries could be represented equally without over or underrepresentation based on geographic area, and by WHO Rabies Statuses: (**A**) represents Status 1 and 2 only, (**B**) represents Status 3 and 4 only, and (**C**) represents all countries.

When applying study eligibility criteria, 113 (47.1%) countries reported surveillance data eligible for inclusion for the testing rate analysis. There were five countries which did not report the type of surveillance system (active or passive), with three countries reporting both systems were in place; the remaining countries reported a passive system or were assumed to be passive based on reporting to a Tier 1 data source. The median number of animals tested annually was 188, with 43.2% domestic animals, 41.7% wildlife animals, and with remaining animals not being differentiated. Data was found for most countries within Europe (42/59), followed by the Americas (28/53), Africa (26/51), and Eastern Mediterranean (9/21); data from Southeast Asia (3/11) and Western Pacific (5/39) were combined due to the limited number of countries within each group that had eligible data (Fig. 2). Countries with the greatest amount of data based on WHO Status were those with Status 1 (42/72) or Status 5 (51/57) (Table 1).

**Table 1:** Testing rates by WHO Status and region.

Grouping (n)	All-animal testing rate, median (IQR)	Domestic animal rate, median (IQR)	Domestic animal rate per dog population, median (IQR)	Wildlife rate, median (IQR)
WHO Rabies Status				
Status 5: No dog rabies (51)	3.4 (0.3–14.3)	0.9 (0.1–2.0)	5.6 (0.9–13.6)	3.5 (0.3–15.3)
Status 5a: Wildlife free (31)	1.3 (0.3–9.9)	0.6 (0.1–1.3)	4.6 (0.5–9.8)	0.7 (0.2–9.5)
Status 5b: Wildlife enzootic (20)	7.8 (2.3–22.4)	1.8 (0.7–5.3)	12.8 (3.5–43.9)	7.8 (1.7–20.1)
Status 4: Controlled dog rabies (8)	4.5 (2.9–13.0)	4.1 (2.3–12.6)	33.1 (18.3–92.7)	0.2 (0.1–0.5)
Status 3: Sporadic dog-transmitted human rabies (5)	8.0 (0.9–31.1)	4.6 (0.6–9.4)	5.8 (< 0.01–14.2)	10.6 (4.3–46.9)
Status 2: Endemic dog rabies (7)	0.5 (0.3–1.1)	0.3 (0.2–0.8)	2.1 (2.0–6.5)	0.3 (< 0.01–0.8)
Status 1: Endemic dog-transmitted human rabies (42)	0.6 (0.1–2.6)	0.4 (0.1–1.9)	3.3 (1.1–17.1)	0.02 (0–0.1)
TOTAL				
Status 1 and 2 (49)	0.6 (0.2–1.9)	0.4 (0.1–1.8)	2.8 (1.1–17.0)	0.02 (0–0.1)
Status 3 and 4 (13)	5.6 (1.9–14.7)	4.6 (0.8–12.1)	23.8 (6.6–60.2)	0.2 (0.1–0.9)
WHO region				
Africa (26/51)	0.2 (0.1–1.4)	0.2 (0.1–1.2)	2.1 (1.1–12.0)	0.01 (0–0.1)
Americas (28/53)	1.2 (0.4–3.9)	0.8 (0.3–4.7)	6.2 (1.9–23.4)	0.1 (0.01–1.0)
Eastern Mediterranean (9/21)	0.6 (0.2–1.6)	0.8 (0.1–2.0)	5.5 (0.4–19.0)	0.03 (0.02–0.04)
Europe (42/59)	6.6 (1.3–17.1)	0.9 (0.2–2.3)	6.1 (1.4–19.6)	3.6 (0.3–18.4)
Southeast Asia and Western Pacific (9/50)	4.8 (0.6–7.4)	3.7 (0.6–5.4)	39.0 (6.6–112.2)	0.4 (0.2–1.1)

All rates are per 100,000 population. N’s within each column may differ based on available data.

### All-animal testing rate (AAHR)

The median AAHR was 1.5 (IQR 0.2–8.1). When stratifying by WHO rabies Status in order of lowest to highest level of control (1–5) the median AAHR were: 0.6 (IQR 0.1–2.6), 0.5 (IQR 0.3–1.1), 0.9 (IQR 0.7–8.0), 4.5 (IQR 2.9–13.0), and 3.4 (IQR 0.3–14.3), respectively (Table 1). In countries with Status 5a the rate was 1.3 (IQR 0.3–9.9) and for Status 5b the rate was 7.8 (IQR 2.3–22.4).

### Domestic animal testing rates (DAHR & DADR)

Among countries with data on domestic animal testing (n = 92), the median DAHR was 0.8 (IQR 0.2–2.8) and DADR was 5.7 (IQR 1.1–24.0). The DAHR and DADR by increasing WHO Status were for Status 1: 0.4 (IQR 0.1–1.9) and 3.3 (IQR 1.1–17.1), Status 2: 0.3 (IQR 0.2–0.8) and 2.1 (IQR 2.0–6.5), Status 3: 4.6 (IQR 0.6–9.4) and 10.6 (IQR 4.3–46.9), Status 4: 4.1 (IQR 2.3–12.6) and 33.1 (IQR 18.3–92.7), and Status 5: 0.9 (IQR 0.1–2.0) and 5.6 (IQR 0.9–13.6) (Table 1). In countries with Statuses 5a and 5b, the rates were 0.6 (IQR 0.1–1.3) and 4.6 (IQR 0.5–9.8), and 1.8 (IQR 0.7–5.3) and 12.8 (IQR 3.5–43.9), respectively.

### Wildlife testing rates (WHR)

Among countries with data on wildlife testing (n = 78), the median WHR was 0.3 (IQR < 0.1–4.3). WHR by increasing WHO Status were for Status 1: 0.02 (IQR 0–0.1), Status 2: 0.3 (IQR < 0.01–0.8), Status 3: 0.01 (IQR < 0.01–5.43), Status 4: 0.2 (IQR 0.1–0.5), and Status 5: 3.5 (IQR 0.3–15.3). In countries with Statuses 5a and 5b, WHR were 0.7 (IQR 0.2–9.5) and 7.8 (IQR 1.7–20.1).

### Threshold calculation

**Figure 3:**
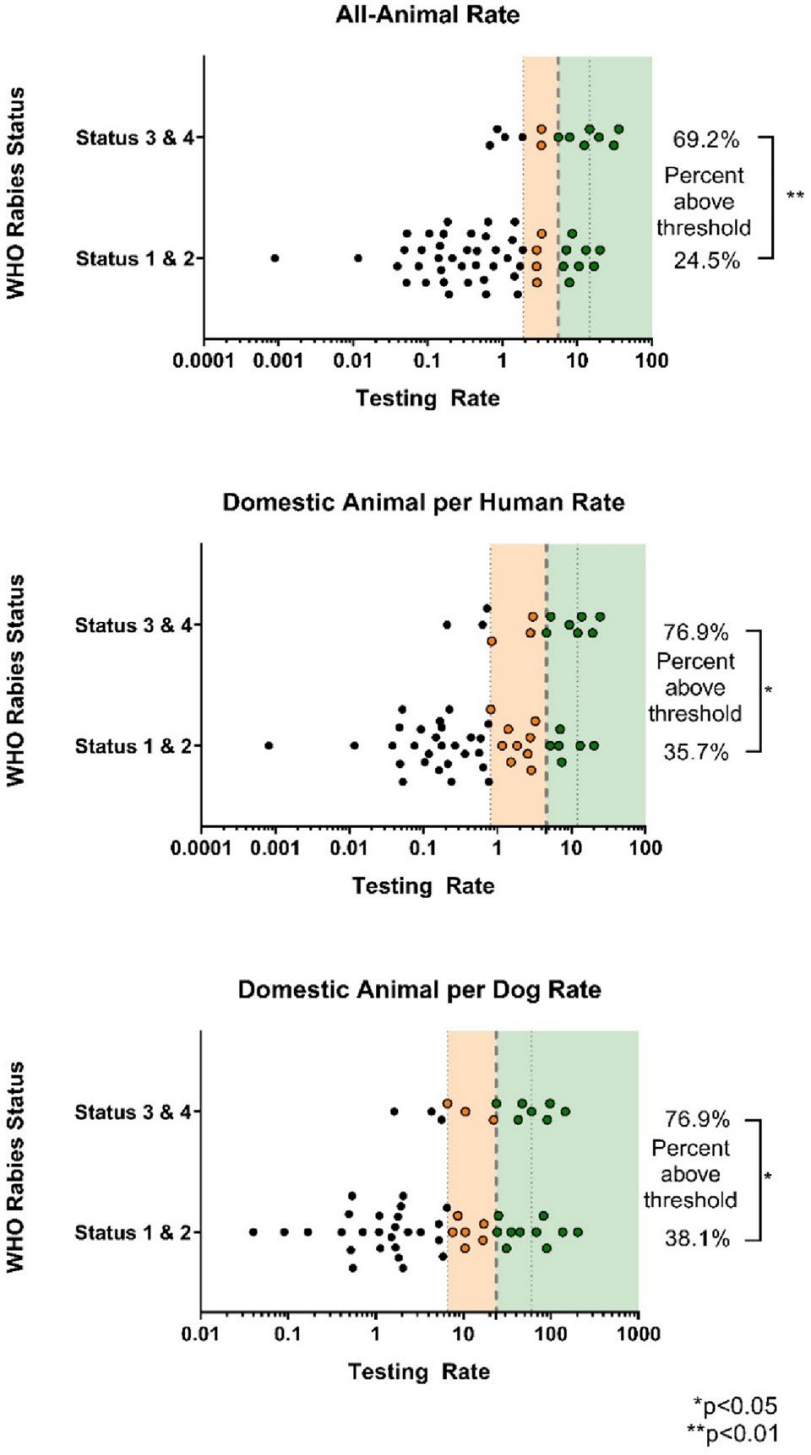
Animal Testing Rate Thresholds. Individual points represent countries with available testing data. The orange section represents any value between the first and second quartile, where the first quartile is the proposed threshold. The green section represents anything at or above the second quartile.

Thirteen countries were listed by WHO as either Status 3 (sporadic dog-transmitted rabies) (n = 5) or Status 4 (controlled dog rabies) (n = 8) and had data that could be used to calculate median testing rates. One country (Ecuador) had reported testing approximately 10,000 cattle yearly as part of active surveillance; therefore, the cattle testing was removed. The median AAHR was 5.6 (IQR 1.9–14.7), DAHR was 4.6 (IQR 0.8–12.1), and DADR was 23.8 (IQR 6.6–60.2) (Fig. 3). The three proposed minimum acceptable testing threshold rates for countries with DMRVV based on the first quartile of peer-derived testing practices are: an AAHR of 1.9, DAHR of 0.8, and DADR of 6.6.

## Discussion

There were clear trends in rabies testing practices reflecting the rabies epidemiology, geographic region, and WHO DMRVV Rabies Status of a country. Notably, rabies testing rates were highest among countries in the final stages of DMRVV elimination (WHO Statuses 3 & 4) and lowest among countries that are endemic of dog-mediated human rabies deaths (WHO Statuses 1 & 2). The elimination of previous testing targets was necessary due to concerns about ethics and interpretation of testing healthy animals. However, this has led to difficulty in applying systematic criteria to evaluate rabies surveillance programs and interpret WHO and WOAH official status programs. Current standards and surveillance repositories report on animals suspected of rabies and tested within a passive surveillance system, i.e., one that relies on testing animals suspect of rabies illness. These international standards exist to ensure uniform reporting of rabies surveillance data, effective monitoring of a rabies surveillance system, and ensure the minimum data elements vital to a program are recorded^7,10^. Testing of animals outside of these recommended methods is less likely to detect cases and is not recommended. Determination of the adequacy of a rabies program includes considerations of multiple factors, including the method of surveillance, testing rates, geographic coverage of a program, number and type of reservoir species, species representation, vaccination rates, among others that are highlighted in the self-declaration verification. By using peer-derived testing rates from countries which are pursuing rabies freedom, this study proposes testing thresholds that can be used as markers of adequate rabies surveillance and as a goal for which rabies endemic countries can strive.

Wide variations in testing rates were observed across eligible countries. This variability was also seen when classifying countries by their WHO Region. This is likely secondary to the diverse social, political, and economic statuses of countries within each WHO region, making interpretation of regional rates complex. Interpretation of regional testing rates was also confounded by the number of countries with missing data; for example, the Southeast Asia and Western Pacific regions have data from only nine of 50 countries. This was a disappointing realization from this study, as many countries within this WHO Region with reported data are in advanced rabies control stages. Due to these disparities in WHO Regional representation, examining rates by WHO Status displays a clearer and less biased description of rabies control efforts.

Countries with WHO Statuses 1 and 2 (endemic dog-transmitted human rabies and endemic dog rabies) had very low testing rates across all four rate calculations, suggesting that lower testing rates are associated with poor rabies control capacity. However, in countries within Statuses 3 (sporadic dog-transmitted rabies) and 4 (controlled dog rabies), the testing rates observed were much higher, reflective of a country with more advanced rabies control capacity and pursuing DMRVV freedom. Testing rates were highest among countries that had eliminated DMRVV (i.e., Status 5),  especially those endemic for wildlife rabies virus variants. This likely reflects a well-established and highly functional rabies control programs but may over-represent testing capacity necessary to document control or elimination of DMRVV. The elimination of DMRVV typically unveils previously unrecognized wildlife reservoirs, therefore, wildlife testing after DMRVV elimination often increases as a result. Therefore, countries with WHO Statuses 3 and 4 were deemed most appropriate to generate minimum acceptable testing thresholds for DMRVV endemic countries. Furthermore, the first quartile of the testing thresholds, which reflects three-quarters of the countries in WHO Statuses 3 and 4, is proposed as the minimum testing threshold to support a claim of adequate surveillance capacity. Countries that are testing at median or higher testing rates and those that exceed more than one of the proposed testing threshold rates are likely to have greater confidence in their surveillance capacities.

The three proposed minimum acceptable testing rates (AAHR of 1.9, DAHR of 0.8, and DADR of 6.6) were standardized for human and dog populations to make comparison across countries more suitable. Ideally, a country with an adequate surveillance program should surpass all three thresholds. However, there are situational circumstances where one or more of these thresholds may not be reasonable to apply. Certain countries or sub-national entities have highly built-up human populations (e.g., communities with numerous high-rise housing complexes); these communities are often associated with lower rates of dog ownership, resulting in high human populations and relatively low dog populations. In these settings, it would be reasonable to expect an adequate surveillance program to fail to reach the AAHR and DAHR testing thresholds while surpassing the DADR threshold. The converse of this may also occur; in areas with low human populations densities and high rates of dog ownership (e.g., rural, pastoral communities), testing rates per human population (AAHR and DAHR) may appear higher while the DADR may appear low. Rabies surveillance programs should be tailored to address the circumstances of the catchment population, which can differ greatly across and within countries; all three threshold rates are important markers of surveillance capacity and should be considered within the context of the human and dog populations to which they are applied.

Twelve countries within WHO Statuses 1 and 2 exceeded the minimum AAHR threshold, 15 exceeded the minimum DAHR threshold, and 16 exceeded the DADR thresholds; some surpassed the medians as well. Many of these countries have published extensively on their commitment to establish rabies control programs, including specific investments in rabies surveillance infrastructure. For example, Namibia has partnered with international experts and WOAH to establish a Rabies Twinning Project focused on improving laboratory-based surveillance systems. South Africa has an officially recognized WOAH Rabies Reference Laboratory, and is one of only 13 countries with such recognition. Philippines is one of just two countries that has received the WOAH Status of an “Official Control Programme for Dog-Mediated Rabies” (the other is Namibia). Thailand received robust political commitment for rabies control from the Royal Princess and currently has one of the highest testing rates outside of the Western Hemisphere. These countries which are exceeding rabies surveillance expectations each have unique experiences that have resulted in their ability to outperform their rabies peer-groups, and they serve as potential case-studies for overcoming poor rabies surveillance infrastructure. As time goes on, it is expected that more countries will move into these categories and these thresholds should be updated regularly (e.g., every 5–10 years) to reflect these changes.

It is important to emphasize that these threshold rates were taken from passive surveillance systems (whether that be from surveillance repository or the literature). For example, one country (Ecuador, WHO Status 4) had a very high testing rate, but it was secondary to testing of approximately 10,000 cattle annually under an active vampire bat rabies surveillance program. A rate by itself is not useful without having clear criteria describing testing eligibility, the method of surveillance (active vs. passive), control measures that have been implemented, geographic distribution of surveillance efforts, and rabies reservoirs found within the country. For this analysis, and interpretation of a testing threshold, we assume that testing data from Tier 1 surveillance repositories was conducted as part of public health or animal health surveillance programs targeting animals with a clinical suspicion for rabies (typically referred to as passive surveillance). Any application of this testing threshold must only be implemented among eligible animals and eligible surveillance systems; the testing of animals with no clinical suspicion for rabies virus infection provides no epidemiologic benefit and in most situations is questionably ethical.

Sharing this type of data on publicly available, international surveillance repositories is the best way to inform rabies control efforts. Open data sharing to publicly available repositories create a forum for monitoring and evaluation of international rabies control efforts and provides empirical evidence to support rabies control policies. Furthermore, WHO- and WOAH-member countries have a legal obligation to report certain health events, including rabies case data. The WHO houses the GHO rabies data portal and provides publicly available summaries and annual reports based on voluntarily submitted national data. However, only 59 of 194 (30.4%) nations report to the GHO rabies portal annually and only one variable (i.e., human deaths) is consistently reported and displayed. By becoming a WHO member country, representatives agreed to report on critical health data in a timely manner; this includes data on rabies cases in humans and animals, among other requested data elements^25^. Countries need to have effective surveillance programs, ensure rabies is notifiable, and report regularly to GHO so that it can be tracked openly. If utilized by WHO-member countries, this publicly available resource can provide significant insight when making decisions to control the spread of rabies and monitor progress towards global initiatives outlined in the NTD roadmap and the UN Sustainable Development Goals.

There are several limitations to this study. The primary limitation is the heterogenous data that was used to synthesize this analysis and prevented analyses of significance; different data repositories are not standardized, and neither is data on ministry websites or in publications. Therefore, only data was included that fit the strict study eligibility criteria to allow for country comparisons and data aggregation. Wildlife reservoirs of rabies virus in many countries is unknown and under-reported. Therefore, analyses of wildlife testing rates are limited outside of Status 5 countries where wildlife reservoirs are largely well-described. However, assessment of any country that has achieved DMRVV freedom should include an evaluation of testing during the years preceding the declaration, where testing would have been expected to be high to ensure the elimination of DMRVV. These limitations necessitate the utility of using testing rates in combination with other parameters to evaluate a country’s overall rabies risk such as laboratory testing capacity, public health support, and rabies endemicity Status. Lastly, the testing thresholds presented here are based on the practices of eligible rabies programs; they are not derived from bio-statistical models or detection probability analyses, as have been used and proposed elsewhere. While these advanced modeling methods can be useful to inform surveillance capacity building, the peer-derived analysis conducted here provides important insight into realistic testing expectations across a range of socio-economic conditions and is supported by empirical evidence of rabies control successes seen in higher testing countries.

This study highlights a new parameter that can be used to examine a rabies control program which can be measured over time and tracked; the authors encourage countries to publicly report rabies data to appropriate international agencies to support evidence-based recommendations and provide insight into global targets (e.g., ZB30).

## Supplementary Information

Supplementary Table S1.

## Data Availability

The datasets generated and/or analysed during the current study are available in publicly available datasets for SIRVERA, GARC, and the European bulletin repository, (SIRVERA - Sistema de Informação Regional para Vigilância Epidemiológica da Raiva (panaftosa.org.br), Home | Global Alliance for Rabies Control (rabiesalliance.org), Rabies - Bulletin - Europe | Rabies Information System of the WHO (who-rabies-bulletin.org)).
