# Supplementary material for: Analysis of available animal testing data to propose peer-derived quantitative thresholds for determining adequate surveillance capacity for rabies
Source: Sci Rep. 2023 Mar 9;13:3986. doi: 10.1038/s41598-023-30984-3 (PMC9998015; doi:10.1038/s41598-023-30984-3)
Supplement: Supplementary file 1 — Supplementary Table S1. [file 41598_2023_30984_MOESM1_ESM.pdf]

Supplements

Table S1: Study Tiers

Study Tier	Tier description	Number of data sources fitting category (N = 127) N (%)	Study end year (range)
1	Regional or national surveillance repositories or annual country report	89 (70.1)	2014–2019
2	Published report in peer-reviewed literature or on a ministry of health site that includes data from the entire country	34 (26.8)	2004–2019
3	One time study examining rabies prevalence or similar objective OR a published report including a region of a country	3 (2.4)	2012–2014
4	Other	1 (0.8)	2014
